# Enrollment Assister Perspectives of a Private Health Insurance Program for Undocumented Immigrants

**DOI:** 10.1001/jamahealthforum.2026.1514

**Published:** 2026-06-05

**Authors:** Christine C. Welles, Maria F. Rodriguez, Chloe Glaros, Elizabeth Boggs, Rachel Peters, Nina Schwartz, Brooke Dorsey, Lilia Cervantes

**Affiliations:** 1 Division of Hospital Medicine, University of Colorado Anschutz Medical Campus, Aurora; 2 Department of Public Health, University of Colorado Anschutz Medical Campus, Aurora; 3 Division of Insurance, Department of Regulatory Agencies, Denver, Colorado; 4 Adult and Child Center for Outcomes Research and Delivery Science Colorado, Aurora; 5 Connect for Health Colorado, Aurora

## Abstract

**Importance:**

In 2023, Colorado implemented the only state-funded marketplace-like US private health insurance program for undocumented immigrants. Community health workers and brokers served as enrollment assisters (EAs) and played a pivotal role in helping clients enroll in insurance plans and navigate the health care system. Understanding the perspectives of EAs is important to inform scalability and implementation as other states consider launching similar state-funded health insurance programs for undocumented immigrants, particularly amid declining federal support for underserved populations.

**Objective:**

To explore the experiences of EAs who supported undocumented immigrants in enrolling in and using private health insurance.

**Design, Setting, and Participants:**

This qualitative study included semistructured interviews conducted between December 2024 and March 2025 in both English and Spanish at EA sites across Colorado. Interviews were audio-recorded, translated, transcribed, and analyzed using thematic analysis. EAs who enrolled undocumented immigrants in private health insurance during at least 2 of the enrollment years from 2023 to 2025 were interviewed.

**Main Outcomes and Measures:**

Themes and subthemes.

**Results:**

A total of 24 individuals (16 [67%] female; 8 [33%] male) participated. Four themes were identified: (1) improved health and reduced reliance on emergency services (limited insurance options; improved health and quality of life; reduced need for emergency services), (2) limited spots, high demand, and need for health literacy education (emotional distress from limited spots amid high need; low-level health insurance literacy and need for education), (3) strong rapport with EAs helps to alleviate fears of deportation (perceived risk to future citizenship from insurance enrollment; community relationships, trust, and rapport), and (4) culturally responsive strategies for resources, processes, and policy (building connection through culturally informed preenrollment resources; enhancing postenrollment support for lasting impact; redesigning the allocation process; strategies for policy improvements to strengthen the framework).

**Conclusions and Relevance:**

In this qualitative study, EAs perceived that the program fills a long-standing gap in health insurance coverage, improving clients’ health and quality of life while reducing emergency use. They also identified challenges related to the limited number of spots and client unfamiliarity with insurance. These findings may inform efforts by other states seeking to develop similar programs for undocumented immigrants.

## Key Points

**Question:**

How do community health workers and brokers, serving as enrollment assisters, describe their experiences in helping undocumented immigrants enroll in the first state-sponsored US private health insurance program?

**Findings:**

In this qualitative study of 24 enrollment assisters, participants perceived that the program addressed a long-standing gap in coverage, improving clients’ health and quality of life while reducing reliance on emergency care. Enrollment assisters also reported challenges, including high demand for the program and limited client familiarity with insurance.

**Meaning:**

These findings suggest that expanding the program and providing culturally tailored educational resources may have benefits for the health and well-being of undocumented immigrants.

## Teaser

This qualitative study assessed the perceptions of enrollment assisters in Colorado’s state-funded, marketplace-like private health insurance program.

## Introduction

An estimated 50% of undocumented immigrants lack health insurance, compared with 8% of US citizens.^1^ This disparity stems largely from their exclusion from nearly all government-sponsored health insurance programs, including Medicare, Medicaid, and Affordable Care Act (ACA) marketplace plans.^1,2^ Many also work in low-wage positions without employer-sponsored health insurance, and even when coverage is available, premiums are often unaffordable.^3^ As a result, undocumented immigrants frequently rely on emergency Medicaid and receive care through emergency departments (EDs). Improving access to preventive and routine outpatient care may reduce avoidable ED use by enabling earlier management of chronic and acute conditions.^4,5^ Consequently, many states are exploring strategies to expand insurance coverage to reduce reliance on costly emergency care.^6,7^

In alignment with this broader policy movement, Colorado implemented the first and only state-funded US marketplace-like program providing private health insurance to undocumented immigrants in 2023. This initiative was well received, with 11 000 available spots filling in less than 2 days in 2024.^8^ Individuals enrolled through this program described no longer delaying care due to cost concerns, seeking care earlier, improving chronic disease control, and decreasing reliance on ED services.^9^ As the only program of its kind in the US, it has drawn national attention as federal support for underserved populations declines.

Community health workers (CHWs) and insurance brokers, collectively referred to as enrollment assisters (EAs), support the program. They play a pivotal role in the program’s success by helping clients enroll, select plans, and navigate the health care system. Previous studies have demonstrated importance of ACA navigators and marketplace assisters,^10,11,12,13,14^ CHWs,^11,15,16,17,18^ and health insurance literacy interventions.^19,20,21,22,23,24^ However, little is known about the experiences and challenges EAs encounter when supporting undocumented immigrants newly gaining access to US health insurance.

As other states consider similar programs, understanding EA perspectives is critical to inform implementation, improve health insurance literacy, and ensure equitable access. We therefore conducted a qualitative study examining the experiences and insights of EAs supporting undocumented immigrants enrolling in private health insurance.

## Methods

### Study Design

Semistructured 1-on-1 interviews were conducted between December 2024 and March 2025 in English or Spanish, based on participant preference. Participants provided verbal consent (including for quotations to be published) and received $50 renumeration consistent with qualitative research norms in Colorado. This study was approved by the Colorado Multiple Institutional Review Board and followed the Consolidated Criteria for Reporting Qualitative Research (COREQ) reporting guideline.^25^

### Setting and Participants

Eligible participants were adult (aged ≥18 years) EAs in Colorado who assisted undocumented immigrants with enrollment during at least 2 of the 3 enrollment years (2023-2025). Participants were recruited via email by the health insurance marketplace in Colorado (Connect for Health Colorado). Purposive sampling using a prescreening form allowed us to identify an equal number of CHWs and insurance brokers.

### Outcome Measures

Demographic data were collected in REDCap version 15.0.28 (Vanderbilt University), including age, race or ethnicity (self-reported from a prescreening form in REDCap), preferred language, country of origin, highest level of education, EA type (CHW or insurance broker), county of residence, county of clients served, languages spoken with clients, and years of participation. An interview guide was developed following a literature review on health insurance access for undocumented immigrants and on the role of health insurance EAs.^10,12,13,26,27,28,29,30,31,32,33,34,35,36,37^ The interview guide posed questions to participants about enrollment processes, challenges, health care navigation, recommendations for improvement, and perceived program impact (eMethods in Supplement 1).

### Data Collection

One physician researcher (C.C.W.) and 1 bilingual researcher (M.F.R.), both with extensive experience in working with undocumented immigrant populations and trained in qualitative interviewing, conducted interviews with participants via video. Interviews continued until thematic saturation (ie, no new concepts or ideas) was reached in each subgroup of EA type (insurance brokers and CHWs).^38^ Certified professional translators translated and transcribed interviews verbatim (Datagain, Inc, and Landmark, Inc). A bilingual team member (M.F.R.) reviewed the transcripts to ensure accuracy.

### Statistical Analysis

Three team members (C.C.W. M.F.R., and C.G., a qualitative analyst), developed an inductive codebook with oversight from a PhD-level qualitative methodologist (B.D.). Using Atlas.ti version 25 (Scientific Software Development GmbH), the team independently reviewed 4 transcripts line by line to refine codes. The finalized codebook was applied to the remaining transcripts, with 20% (2 transcripts from CHW interviews and 2 transcripts from insurance broker interviews) triple coded to ensure calibration. Using content analysis, 5 of us (C.C.W., M.F.R., C.G., B.D., and L.C.) grouped codes, identified conceptual links, and reached consensus on themes and subthemes. Coding disagreements were resolved by consensus meetings. Preliminary analysis did not show a difference between CHW and insurance broker subgroups (based on the absence of divergent codes or theme-level differences), so the groups were combined for the final analysis. Triangulation (multiple researchers independently coding transcripts and then comparing themes) was used to ensure that the findings reflected the full range and depth of the data. Member checking was not conducted to avoid additional burden on participants given the controversy in the efficacy of this technique.^39^

## Results

A total of 36 individuals filled out the initial prescreening form. Information on age was not collected. Reasons for nonparticipation included no response (n = 7), ineligible due to only enrolling for 1 year (n = 4), and declined participation (n = 1). We conducted semistructured interviews with 24 EAs including 11 insurance brokers, 12 CHWs, and one EA from a community clinic (Table 1). These 24 EAs (16 [67%] female; 8 [33%] male) were recruited from 23 different sites, including 12 community health centers and 11 brokerage firms. A total of 16 EAs (67%) were women and 8 (33%) were men. A total of 18 interviews were conducted in English (75%) and 6 in Spanish (25%). Mean (SD) interview duration was 45.7 (7.7) minutes. Four themes were identified. Table 2 summarizes themes, subthemes, and illustrative quotations. The Figure shows thematic schema illustrating the themes.

**Table 1.** Participant Characteristics

Characteristic	Participants, No. (%)
Type of enrollment assister	
Community health worker	12 (50)
Broker	11 (46)
Enrollment assister from community health clinic	1 (4)
Sex	
Male	8 (33)
Female	16 (67)
Language in which interview was conducted	
English	18 (75)
Spanish	6 (25)
Race and ethnicity^a^	
Asian	1 (3)
Black or African American	2 (8)
Hispanic	14 (58)
Multiracial	1 (3)
White, not Hispanic	5 (21)
Prefer not to answer	1 (3)
Country of origin	
US	11 (46)
Mexico/Latin America	5 (21)
South America	5 (21)
Other^b^	3 (13)
No. of years at current job, mean (SD)	5.5 (3.6)
Location of participants enrolled	
Urban	17 (71)
Rural	5 (21)
Both urban and rural	2 (8)
Live in different county than enrolling	
Yes	2 (8)
No	21 (88)
Did not answer	1 (4)
Educational level	
High school diploma or GED	2 (8)
Some college, no degree	5 (21)
Associate’s degree	3 (13)
Bachelor’s degree	5 (21)
Master’s degree	6 (25)
Professional degree	3 (13)
Years of experience enrolling in the program	
2	6 (25)
3	18 (75)
Languages spoken with participants	
English	3 (13)
Spanish	2 (8)
English and Spanish	14 (58)
English and other^c^	4 (17)
English, Spanish, and other	1 (3)

Abbreviation: GED, general education development.

^a^ Self-reported from a prescreening form in REDCap.

^b^ Other included Somalia, Burundi, and China.

^c^ Other category was self-selected by patients.

**Table 2.** Themes and Subthemes With Illustrative Quotations

Themes and subthemes	Illustrative quotations^a^ (participant No.)
**Theme 1: Improves health and reduces reliance on emergency services**	
Limited insurance options	Sadly, when they saw the prices of health insurance, they didn’t know whether it was better to forgo treatment or go into debt paying for insurance. (34)
	Ninety-six, 97% of the population that we serve, don’t have or didn’t have a health program. They didn’t qualify because of immigration status. So, the way our population used medical services was only for emergencies. There was no kind of prevention or check-up, or being aware of their health, but it was more of a reaction to an emergency, to an accident, to a flu, to anything that came up, so the emergency departments were full. When the emergency departments fill up, the costs are higher for the hospitals and for the state when they can’t pay. Generally, these are families that are below the poverty line. (15)
	Unfortunately, it was a very sad topic because when they came with the motivation of, “I was told you could help me get insurance,” I would ask them the necessary questions, and when they told me they didn’t have documents, I had to say that unfortunately, there was no program for them, that it was only for people who had documents and met certain requirements. The good thing is that it still exists, but I also think that, so far, of all the states I work in, Denver is the only one that offers this kind of benefit. (25)
Improved health and quality of life	These people work hard, and they’re always worried about something. When OmniSalud came along, that was wow ‘cause, now, people have the resources to treat diabetes or the kidney problems or heart problems or many other—cancer, ‘specially breast cancer for women. It’s quite high. Now, they have the option. They have the opportunity to get treated and to get treated well. (27)
	They come here, they are afraid to go to a hospital. They’re suffering a lot of issues, and definitely once they have the OmniSalud, they have that, their burden was relieved. Not only just for physical. They need to go to the doctor and a hospital thing, but it’s also mentally, is for their life—stress is gonna resolved so heavily. It’s a great help for their life quality, is great health to make them feel this is their home. They’re wanting to building our community. They want to help us a lot. (35)
Reduced need for emergency services	Obviously a significant amount of people are happy that they have access to the OmniSalud program, because quite honestly, most people are working multiple jobs, and to pay for insurance is pretty impossible, especially with the cost of living…. Across the board, you’re going to hear from a lot of interviews that this is—this is a program that has saved lives, because we’re seeing a reduction in ER [emergency department] visits, and we’re seeing an increase in primary care visits, to where they can actually access a primary care doctor and keeping the emergency departments free. (5)
	I think putting it into perspective of if you have—I mean actually probably all states now have a high population of immigrants coming in and looking at the data from their ERs [emergency departments] and how many immigrants are coming in and utilizing the ER as a swinging door. As unfortunately, trying to control costs that way, we’re all feeling the effects of that. (33)
**Theme 2: Limited spots, high demand, and need for health literacy education**	
Emotional distress from limited spots amid high need	For this year, people were disappointed. People came in at 4:00 or 5:00 in the morning. It’s not something we recommended people to come that early, but people said, “No, I really need this resource. I don’t have insurance, and insurance is so expensive. I need it because I take medication,” or “I have a chronic condition,” or “I have an appointment,” or “I have a surgery coming up, and I really can’t afford it.” We had a big line of people. Our office opens at 8:00 in the morning. Our parking lot was full. We had a long, long line of people that were just waiting outside, and it was cold. We helped as many people as we could, but we had to turn some people away. (4)
	“I really needed it. I was going to get a surgery.” I mean, I was the bearer of bad news. I was the bad guy. I have people who were mad at me and said, “I’m just going to find somebody else because you promised me.” I was like, “No, I did not promise you anything.” It wasn’t my fault. I tried. Believe me, I tried…. you’ll always have people who just want to take it out on somebody. I guess I did the best I could. (30)
	A lot of them are dialysis, kidney transplants, things of that nature, and they didn’t make it and you have nothing to offer. (28)
Low-level health insurance literacy and need for education	Sadly enough, the [insurance carrier] was new, so they went to urgent care, paid out of pocket because they couldn’t verify his insurance…. The first thing was I told the urgent care center, what right do you have to take someone’s money up front when they give you proof of insurance? … so I had to appeal the claim, resubmit the claim again because it was wrong. (28)
	Part of it is after you receive [your insurance card], you have to go online on the [insurance carrier’s] website. I believe you have to do a quick registration to activate your plan. She doesn’t speak English, and also she’s not very techie. She can’t navigate to the websites herself. She said, “I received the card. What do I do next?” Then it was like, “Okay, let’s get together. Let’s set a time. Let’s figure this out so that we can help.” (4)
**Theme 3: Strong rapport with enrollment assisters helps to alleviate fears of deportation**	
Perceived risk to future citizenship from insurance enrollment	Yeah, I think that [deportation] was also, again, a fear for them too. We ended up having to do a focus group where we had a lawyer come in and explain public charge and provide information on how this was being impacted to make sure that folks really understood that this is something that was legal and they were okay to take these programs….That’s how we mitigated that fear. (21)
	…there had never been something like this for the undocumented immigrant community. Typically, this community only has access to discounts at clinics, but they were also facing the challenge that many clinics weren’t offering those discounts or were limiting them, and this was becoming a challenge for them to find medical assistance. Without a status, the chances of having access were very low, and many times they prefer not to go to a clinic or a place out of fear, or because they simply think they can’t access medical health services without a status. (9)
	Exactly, because insurance does have a cost, so their fear was, “If they’re going to charge me later, then it will be more problems than benefits.” Yeah. It inhibited. It did inhibit some applications. Because they’re afraid. (35)
Community relationships, trust, and rapport	I think it does come down to organizations that are truly living with integrity when it comes to customer service of clients, not just during health care coverage, but I think in general, when it comes to any access to their services and resources. Some community members have, and still have, an issue with our county human services office because they don’t feel like they’re treated as humans. They’re just treated as the next number that comes in. Even when my own organization was stretched thin, we always came back to, we want people to walk away being heard and feel respected and also feel compassion toward their needs and not that they’re a burden. (1)
	…they trusted us. Maybe it’s because sometime they saw us in different events. We do community events, health fair clinics, so they were familiar with our face and our work, so they trusted us. (11)
	Sometimes you create a relationship between those clients because they get to know you, because they know that you help ‘em out with something. How about these patients, I create a relationship, they talk to me how their health condition is, what they need. (3)
**Theme 4: Culturally responsive strategies for resources, processes, and policy**	
Building connection through culturally informed pre-enrollment resources	I think as for the future, it might be helpful to have that plan resource page that just talks about the different plans because I’m not really sure if you have worked with assisters or if you are an assister yourself, but it’s quick. You have to do this quickly because these spots really get filled really quickly. It will definitely save time for people to look at the different options so that when we get to that page, they’re like, “Oh, I want this one.” (4)
	I think if it was videos that will talk about the plans, the benefits of having Salud [OmniSalud] or having health insurance, this will help out. (3)
Enhancing postenrollment support for lasting impact	I think that maybe an educational page so that they can understand what a deductible and a co-insurance is. Just the basic wording. Because from my experiences that’s not a word that they would use in Spanish. That’s not how they refer to things with their health care. Starting with the basics of even knowing what each of those mean. Second, maybe putting it into a little flowchart to show what would happen like if you were to go into the emergency department what your charge is. What happens to the deductible? As that progresses, where will you end up with that cost? It would be helpful. Then just a very brief summary in Spanish or whatever language of what their plan entails. (36)
	I think if we had a universal tool from the state that explains, “Hey, this is the health insurance that you just received.” It shouldn’t be too hard to do it because, again, these are all standardized plans, so it’s like, “This is what you got.” It’s just that you got it from A, B, or C, and now this is how you can get preventative care here, or this is how you can get—this is the app. Having that standardized, and that way, we can actually share that so that they know, I think would be super helpful. (21)
	Try to do your best to explain not only the benefits of the insurance plan that you’re getting but also the limits; know those. Mainly, for us, it’s that network. On this plan, you can only go here or only go there. If you have questions, make sure you call your insurance. Make sure that they’re under there. Just being really thoughtful in how you explain how to use that insurance policy that you have. Just so that there’s not surprises, and so that the people who get it actually get the benefit of having insurance. (8)
Redesigning the allocation process	If I had a magic wand, what I would do is that I would try to inject more money into the program. Because the program works, I like it, it’s great for our community. The only thing that didn’t work this year was the technology...the page, the registration, and the system. It’s really bad, I mean, it didn’t work at all this year. (15)
	That’s what I would say is more slots and just ensuring that the platform is ready for an onslaught of enrollment. Because I think because open enrollment is typically so long outside of OmniSalud, they don’t get all the hits in one day. (31)
	People like to know immediately because it’s a lottery. They want to know whether or not they made that lottery. Yeah. I mean, I like the implementation of trying to keep people enrolled that have current medical needs that are going from year to year. I think that’s a great idea. (28)
Strategies for policy improvements to strengthen the framework	…and considering maybe a sliding scale system, instead of a, “you qualify at 100% or you don’t qualify at all,” is the ability to develop a sliding scale system where people might be able to pay a little. I’m sure many people would be willing to pay something instead of just being completely left out. (34)
	The funding, the limit on spots, is due to the funding that’s available and how they have to get the funding because of the residency status. Of the citizen’s status of the people that are getting the assistance. If we could somehow get more money so there would be more spots, that would be great. (8)
	…that’s been the biggest feedback from community is the spots go by so fast and not everyone is able to enroll. It would be great if it worked similar to Medicaid where it’s just available for community without having a restricted set of number that can apply. (1)

^a^ Participant quotations are presented verbatim from the interview transcripts.

**Figure.**
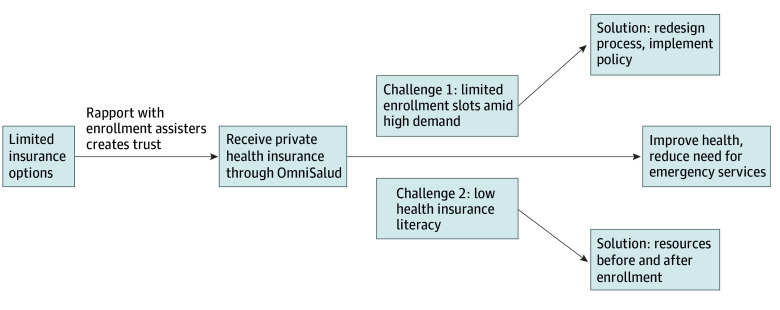
Thematic Schema

### Theme 1: Improves Health and Reduces Reliance on Emergency Services

#### Limited Insurance Options

Prior to the program, EAs felt distress informing clients they had no health insurance options. They felt helpless and frustrated as they witnessed families face challenges, including clients with serious illnesses who were forced to choose between forgoing treatment or incurring debt. Some of their clients had lived in the US for decades without ever seeing a health care professional. EAs described that clients feared becoming ill: “There’s no way I can get medical help; it’s too expensive.” Many clients were reported to worry about potential injury, saying, “I can’t risk getting hurt or injured” (participant 12). Without insurance, EAs shared their clients often forewent preventive care and relied on the ED.

#### Improved Health and Quality of Life

EAs described how health insurance profoundly transformed their clients’ lives. Many clients had endured chronic and painful conditions for years without care. They felt peace of mind knowing their clients could seek care. One EA recalled, “I remember seeing the immediate relief she felt knowing that she has health insurance” (participant 1). When EAs learned of individuals who were struggling with medical conditions, they often reached out proactively to enroll them. With insurance, clients received care for conditions such as heart disease, diabetes, and cancer. They had access to specialty care, preventive care, surgical services and could afford essential medications. Some even received life-saving organ transplants.

#### Reduced Need for Emergency Services

EAs reflected on the broader health care system benefits of the program. They noted that without insurance, clients are forced to use the ED like a “swinging door” (participant 33) and how that in turn increased overall health care costs. EAs perceived the program improved health care use by decreasing reliance on emergency services and increasing use of preventive and primary care services. One EA said, “An ounce of prevention is worth a pound of cure … We let a population of people in the United States get profoundly ill … making it more expensive to help them than it does to try and prevent that in the first place” (participant 7).

### Theme 2: Limited Spots, High Demand, and Need for Health Literacy Education

#### Emotional Distress From Limited Spots Amid High Need

The limited spots, which were allocated on a first-come, first-served basis, resulted in long lines early in the morning, with no guarantee of obtaining a spot. EAs reported clients had to take time off from work, pay for parking, and stand in the cold for hours, yet still sometimes did not receive a spot. The high volume created chaotic enrollment environments, and at one point, the surge in demand caused a systemwide technical glitch that prevented sites from enrolling. EAs found it incredibly difficult to have to bear the burden of delivering bad news, exclaiming, “…[T]hat’s heartbreaking. Hey, I mean, I tear up thinking about it because there’s so many clients that have medical needs and knowing that they didn’t make it” (participant 28). EAs also raised concerns about fairness of spot allocation and lack of prioritization of those with medical needs.

#### Low-Level Health Insurance Literacy and Need for Education

EAs emphasized that a large part of their role was providing health insurance education to their clients. They shared that clients had little to no understanding of how health insurance in the US works because it is not offered in their home countries. One EA shared, “Because there is at least a couple of people who got their insurance card and they said, ‘Okay, is this a debit card? What do I do with this?’” (participant 5). EAs educated their clients on the basics of health insurance as well as the importance of using in-network facilities and receiving preventive care. They encouraged their clients to return for assistance if they had difficulty with health system navigation after receiving health insurance. They also made calls on the client’s behalf, obtained interpretation services, managed unexpected out-of-network bills, and advocated for reimbursement when billing errors occurred.

### Theme 3: Strong Rapport With EAs Helps to Alleviate Fears of Deportation

#### Perceived Risk to Future Citizenship From Insurance Enrollment

EAs described clients often had fears about deportation and incurring public charges when enrolling. One EA noted, “I think everybody has the same concern about it. How will it affect the immigrant people that might one day have a legal status? Will it have an impact on their eligibility to become documented, or will, at one point, it be used as information to track them down?” (participant 22) They reported these fears have recently intensified with the new US administration stance on immigration. EAs cultivated trust with their clients and were able to mitigate these fears by reassuring them that their information was protected and would not be shared. Some community-based organizations organized events where lawyers spoke to clients about their legal rights and protections.

#### Community Relationships, Trust, and Rapport

EAs emphasized that treating clients with dignity, compassion, and respect was central to their work, countering a system that often made immigrants feel dehumanized. They emphasized that their role went beyond processing applications; it involved listening, empathizing, and ensuring that clients felt genuinely cared for. Trust was built over years of consistent involvement in the community, personal connections, and culturally responsive communication. EAs remained a resource after enrollment, always available for questions or concerns. Many clients returned year after year for reenrollment because they felt supported and respected. EAs found this connection deeply fulfilling and expressed pride in serving their communities, knowing their work not only helped individuals access insurance but also advanced the broader goal of equity and inclusion in health care. An EA said, “It’s great to get to know a lot of them and be involved in the community that’s outside of what we all typically do. I feel I’m helping in a good way with the progression of the United States and making sure we all have an equal, fair opportunity to have health care no matter where we come from” (participant 36).

### Theme 4: Culturally Responsive Strategies for Resources, Processes, and Policy

#### Building Connection Through Culturally Informed Preenrollment Resources

EAs emphasized the need for clear and accessible preenrollment resources to help EAs and clients make faster, more informed decisions during enrollment. They recommended offering culturally informed descriptions of health insurance options in multiple languages and formats (videos, handouts, graphics, presentations) to compare plans. One EA explained, “We want to educate them [clients], so they have more precise and relevant information, and above all, avoid confusion” (participant 9). Some suggested hosting workshops or seminars to ensure understanding, while others highlighted the need for more support in rural areas.

#### Enhancing Postenrollment Support for Lasting Impact

EAs emphasized the need for postenrollment education regarding how to use insurance, such as explaining benefits, deductibles, copays, networks, and optimal health care use. They suggested distributing this information through videos, professional presentations, flyers, emails, and visual aids, with content tailored to each community with a range of literacy levels. EAs emphasized that because many clients are unfamiliar with the US health care system, it is essential to present information in culturally and linguistically appropriate ways. One EA explained, “They don’t come from a healthcare system that runs like we do. Keeping something very basic would be very helpful in explaining that to them” (participant 36).

#### Redesigning the Allocation Process

EAs suggested redesigning the system to ensure appropriate distribution of the limited spots. Suggestions for allocation of the spots varied, including first-come, first-served systems, lotteries, renewal priority for previous enrollees, and prioritization of individuals with chronic or high medical needs. One EA shared, “…I would include anyone who has proof of a medical appointment or a doctor’s diagnosis that needs surgery… I would much rather prioritize that the people who enter this program, knowing it’s limited, are people who will actually use it” (participant 12). Technical improvements were also widely recommended, such as preparing for high demand to prevent website crashes during peak enrollment periods, adding real-time support, enabling autopopulation of family data, and allowing for account creation without email.

#### Strategies for Policy Improvements to Strengthen the Framework

EAs called for increased funding and expansion of the program to meet the overwhelming need for affordable insurance. One EA shared, “What I would do is try to inject more money into the program. Because the program works, I like it, it’s great for our community” (participant 15). Others suggested introducing a sliding-scale payment model, so those who were unable to obtain a spot are not left with unaffordable premiums.

## Discussion

In this qualitative study of the first state-sponsored US marketplace-like program providing private health insurance to undocumented immigrants, EAs perceived that the program improved clients’ health and quality of life while reducing use of emergency care. They built trust through sustained community engagement and mitigated deportation concerns. Key challenges included limited enrollment spots and low health insurance literacy. To address these challenges, EAs recommended providing culturally tailored educational resources and expanding the program to increase access.

As federal support for underserved populations declines, states are alternative strategies to support these communities. As the only state-sponsored US program providing private health insurance to undocumented immigrants, this program serves as a model for other states seeking to implement similar legislation.

Undocumented immigrants face substantial barriers to health care access, including fear of deportation^40,41,42^ and limited access to health insurance.^27,30,40,43^ EAs described feeling helpless before the program, as many undocumented clients had no insurance options and delayed treatment for serious conditions due to fear of the cost, often relying on the ED. EAs perceived that gaining insurance greatly improved clients’ health and quality of life, providing relief and access to outpatient primary and specialty care. Although clients initially feared deportation or negative consequences for future citizenship, EAs reported that trusted ongoing relationships helped alleviate those concerns to support enrollment.

A key finding of our study was the need for health insurance education to help clients use private health insurance effectively. Undocumented immigrants face unique challenges with navigating the US health care system, including limited English proficiency,^40,44,45^ low-level health literacy^46,47,48,49^ and low-level health insurance literacy.^40,50,51^ EAs reported that many clients sought guidance on what to do with their insurance cards once they arrived in the mail, reflecting their unfamiliarity with both the US health care system and private insurance. EAs emphasized the need for language and culturally concordant resources, both before and after enrollment. These findings highlight the need for structured, community-based interventions to improve general and insurance-specific health literacy. In our study, EAs went beyond enrollment, providing ongoing support by answering questions and helping clients navigate the system. Although this support was not formally defined within their role and often depended on clients reaching out, it functioned as a critical bridge between coverage and meaningful access to care. This pattern suggests a structural gap in program design, indicating that without dedicated postenrollment support, obtaining insurance does not reliably translate into effective use. Future interventions could formalize postenrollment responsibilities by including proactive outreach to ensure members can effectively use their insurance and navigate any challenges that arise.

EAs also highlighted the high demand for a limited number of available slots, which led to concerns about scarcity and appropriate distribution. They expressed differing opinions on how to prioritize enrollment. Some favored giving priority to current clients, others to those with the greatest medical need, while some supported a lottery system or maintaining the first-come, first-served approach. Applying the Accountability for Reasonableness framework could help ensure that decisions about prioritization are grounded in ethical principles, thereby promoting fair, transparent, and legitimate resource allocation.^52,53^ Despite these differences, all EAs agreed that the program should be expanded to meet the high demand and extend benefits to more people. Several also suggested adopting a sliding-scale model, allowing client contributions to be reinvested to create additional spots. They also recommended strengthening the technology infrastructure to handle high volumes of simultaneous enrollments and prevent system crashes.

### Limitations

Our study has limitations. Participants were recruited from Colorado; generalizability to other settings is unknown. Interviews were only offered in English and Spanish; therefore, perspectives of EAs who did not speak these languages may not have been captured. Additionally, social desirability bias may have caused EAs to censor negative perspectives.

## Conclusions

In this qualitative study, EAs perceived this state-sponsored marketplace-like program which provided private health insurance to undocumented immigrants improved the health and well-being of undocumented immigrants while reducing reliance on emergency care. EAs provide critical support in connecting undocumented immigrants to private health insurance. These findings may inform efforts by other states seeking to develop similar programs amid declining federal funding for health insurance for underserved populations. Future efforts are needed to design community-based interventions to improve general health literacy and health insurance literacy to ensure optimal use of such programs.

## Notes

Supplement 1. **eMethods.** Interview Guide

Supplement 2.
Data Sharing Statement
