# Supplementary material for: Enrollment Assister Perspectives of a Private Health Insurance Program for Undocumented Immigrants
Source: JAMA Health Forum. 2026 Jun 5;7(6):e261514. doi: 10.1001/jamahealthforum.2026.1514 (PMC13241950; doi:10.1001/jamahealthforum.2026.1514)
Supplement: Supplement 1. — eMethods. Interview Guide [file jamahealthforum-e261514-s001.pdf]

Supplemental Online Content

Welles CC, Rodriquez MF, Glaros C, et al. Enrollment assister perspectives of a private health insurance program for undocumented immigrants. *JAMA Health Forum*. 2026;7(6): e261514. doi: 10.1001/jamahealthforum.2026.1514

eMethods. Interview Guide

This supplemental material has been provided by the authors to give readers additional information about their work.

© 2026 Welles CC et al. *JAMA Health Forum*.

eMethods. Interview Guide

1. Can you tell me a little bit about your role and organization?
    a. Probe – community that they serve

2. What were your thoughts when you first heard about Omnisalud before enrolling people into the program?
    a. Probe – benefits
    b. Probe – downsides, challenges, concerns
    c. What did you hear from participants or colleagues about the program? (probes" benefits, concerns)

3. Can tell me about your experience enrolling people into the Omnisalud program?
    a. What worked well about the enrollment process?
    b. What didn't work well about the enrollment process?
    c. Did you have experience with anyone not able to enroll?
    d. What years were you involved in enrollment (Nov. 2022, 2023, 2024)
        i. If multiple years:
            1. How did enrollment differ each year? (1st year, vs limited re-enrollment spots, vs this year)
            2. What was the same about enrollment each year? (same barriers? Not having to learn something new)
            3. Probes: In November of 2022, people were enrolled over 5 weeks, and in November 2023 slots filled within a day and a half. What was that experience like?
    e. What did it feel like for you as an enrollment assistant to have to tell people the spots were all filled?

4. Did people enrolling ask you for assistance in choosing which health insurance plan to enroll into?
    a. **If yes,** what was that experience like for you? (e.g., easy or difficult)
        i. What type of information or advice did you provide them with to choose a plan?
            1. How did you obtain the information that you provided them with?

© 2026 Welles CC et al. *JAMA Health Forum*.

            2. Can you provide an example?
        ii. From your perspective, are there any materials, trainings, or other ways to give enrollers more tools to help people choose their plan?
    b. **If no,** why do you think people didn't ask for assistance in choosing their plan?

5. From your perspective, what has the overall experience for patients/clients been with Omnisalud? (positive or negative).
    a. Can you give some examples?

6. From your perspective, after participants received their Omnisalud health insurance, has access and use of medical care changed?
    a. **If yes** – how?
        i. (positive or negative change, what kinds of changes). Can you give an example?
        ii. Probe: Change in the number of preventative health screenings? PCP visits? Access to specialty care? ED visits? Hospitalizations?
    b. **If no,** why don't you think the Omnisalud program has changed access and use
        i. Can you give an example?

7. Can you tell me about your experience helping participants use their new health insurance and navigate the healthcare system? (**if no experience**, why not? Who do they think patients go to for help navigating)
    a. What things have worked well in helping participants start to navigate their Omnisalud health insurance?
    b. What barriers are there to participants using their Omnisalud health insurance?
        i. Probe: What questions are you most frequently asked by participants?
        ii. Probe: What barriers / concerns are participants encountering most frequently?
        iii. Probes: e.g. Language barriers, general health literacy, general health insurance literacy, difficulty navigating US health system, not sure if providers and tests are covered by their new insurance or how much they will cost, how to set up appointments
    c. What solutions have you found to help participants navigate their care?

© 2026 Welles CC et al. *JAMA Health Forum*.

8. Are you aware of any facilitators or other helpful factors Omnisalud participants have reported when trying to use their insurance?
    a. Probes: e.g. Family fluent in English, family with good health literacy, health insurance literacy, help from community partners in navigating US healthcare system, whether system has integrated care, help from health care staff

9. What resources or organizations do you use or point the Omnisalud enrollees to when they have questions? (videos, handouts, social media, other organizations)
    a. Are there other resources or organizations that enrollees are turning to?
        i. Probes: e.g. Community health organizations? Their doctor's office? Number on back of card? Do they know who to go to for specific questions?

10. Are there any additional resources that participants need? (probes: resources that don't exist/aren't available)
    a. What do you see as the most effective way to deliver resources to Omnisalud participants?
        i. Probe – such as resources that would improve enrollment, healthcare utilization and/or health literacy?
    b. What kinds of organizations should deliver these resources?
        i. Probe: organizations with established trust, history of serving the Latino community, newcomers to the United States, history of health justice work….

11. Are there ways the Omnisalud program could be improved to make it easier to…
    a. enroll people in the program?
    b. enable people to use their insurance and to navigate the healthcare system?
        i. Probe: e.g. educational materials [videos, talks, flyers, CHW support, peer support, informational packets, other]

12. What thoughts do you have on how we could improve enrollment and the experience for people into OmniSalud?
    a. Probe – what would help you?
    b. Probe – what would help people enroll?

© 2026 Welles CC et al. *JAMA Health Forum*.

c. Probe – what would help people navigate and use their new insurance?

13. Is there anything else we haven't talked about yet, that you want to share about your experience (good/bad/general) as an enroller into OmniSalud?

© 2026 Welles CC et al. *JAMA Health Forum*.
