# Supplementary material for: Enrollment Assister Perspectives of a Private Health Insurance Program for Undocumented Immigrants
Source: JAMA Health Forum. 2026 Jun 5;7(6):e261514. doi: 10.1001/jamahealthforum.2026.1514 (PMC13241950; doi:10.1001/jamahealthforum.2026.1514)
Supplement: Supplement 2. — Data Sharing Statement [file jamahealthforum-e261514-s002.pdf]

# Data Sharing Statement

Welles. Enrollment Assister Perspectives of a Private Health Insurance Program for Undocumented Immigrants. *JAMA Health Forum*. Published June 05, 2026.
doi:10.1001/jamahealthforum.2026.1514

**Data**
**Data available:** No

**Additional Information**
**Explanation for why data not available:** Because of the sensitive nature of the data (interviews with undocumented immigrants)
